# VBP1 modulates Wnt/β-catenin signaling by mediating the stability of the transcription factors TCF/LEFs

**DOI:** 10.1074/jbc.RA120.015282

**Published:** 2021-01-13

**Authors:** Haifeng Zhang, Xiaozhi Rong, Caixia Wang, Yunzhang Liu, Ling Lu, Yun Li, Chengtian Zhao, Jianfeng Zhou

**Affiliations:** 1 Key Laboratory of Marine Drugs, Chinese Ministry of Education, School of Medicine and Pharmacy, Ocean University of China, Qingdao, China; 2 Laboratory for Marine Drugs and Bioproducts, Pilot National Laboratory for Marine Science and Technology, Qingdao, China; 3 Institute of Evolution and Marine Biodiversity and College of Marine Biology, Ocean University of China, Qingdao, China; 4 Laboratory for Marine Biology and Biotechnology, Pilot National Laboratory for Marine Science and Technology, Qingdao, China

**Keywords:** VBP1, pVHL, TCF/LEF, Wnt/β-catenin signaling, Wnt signaling, T-cell factor (TCF), transcription factor, zebrafish, cell biology

## Abstract

The Wnt/β-catenin pathway is one of the major pathways that regulates embryonic development, adult homeostasis, and stem cell self-renewal. In this pathway, transcription factors T-cell factor and lymphoid enhancer factor (TCF/LEF) serve as a key switch to repress or activate Wnt target gene transcription by recruiting repressor molecules or interacting with the β-catenin effector, respectively. It has become evident that the protein stability of the TCF/LEF family members may play a critical role in controlling the activity of the Wnt/β-catenin signaling pathway. However, factors that regulate the stability of TCF/LEFs remain largely unknown. Here, we report that pVHL binding protein 1 (VBP1) regulates the Wnt/β-catenin signaling pathway by controlling the stability of TCF/LEFs. Surprisingly, we found that either overexpression or knockdown of VBP1 decreased Wnt/β-catenin signaling activity in both cultured cells and zebrafish embryos. Mechanistically, VBP1 directly binds to all four TCF/LEF family members and von Hippel-Lindau tumor-suppressor protein (pVHL). Either overexpression or knockdown of VBP1 increases the association between TCF/LEFs and pVHL and then decreases the protein levels of TCF/LEFs via proteasomal degradation. Together, our results provide mechanistic insights into the roles of VBP1 in controlling TCF/LEFs protein stability and regulating Wnt/β-catenin signaling pathway activity.

The evolutionarily conserved canonical Wnt/β-catenin pathway plays a pivotal role in various biological processes, such as cell proliferation and cell fate determination during embryonic development and tissue homeostasis (1, 2, 3, 4). Misregulation of Wnt/β-catenin signaling is associated with many human diseases, such as human birth defects, cancer, and degenerative disorders (2, 3, 4, 5, 6). In the absence of Wnt ligands, cytoplasmic β-catenin is phosphorylated by a multicomponent degradation complex, consisting of the scaffolding protein Axin, the tumor-suppressor adenomatous polyposis coli, casein kinase 1 (CK1), and glycogen synthase kinase 3β (GSK3β), which results in proteasomal degradation of β-catenin. In the nucleus, TCF/LEF acts as a transcriptional repressor by interacting with the transducin-like enhancer of split (TLE)/Groucho protein (7, 8). In the presence of Wnt ligands, stabilized β-catenin accumulates and translocates to the nucleus to form a complex with TCF/LEFs, leading to the transcriptional activation of Wnt target genes (3, 4). Therefore, the outcome of Wnt/β-catenin signaling is controlled by the complex of β-catenin and TCF/LEFs. As key components of Wnt/β-catenin signaling, TCF/LEFs are conserved among different species. A single TCF/LEF gene is found in *Drosophila* and worm genomes, whereas vertebrate genomes harbor four TCF/LEF genes (9). It was reported that nuclear concentrations of both TCF/LEFs and β-catenin are important for establishing an optimal β-catenin:TCF ratio, which is crucial for the activity of Wnt/β-catenin signaling in *Xenopus* and *Caenorhabditis elegans* (10, 11). Thus, Wnt/β-catenin signals may regulate gene expression by dynamically controlling the concentrations of TCF/LEFs and β-catenin in the nucleus. In contrast with the well-understood posttranslational regulation of β-catenin, regulation of TCF/LEF proteins awaits further elucidation. Studies have shown that the mitogen-activated protein kinase–related protein kinase Nemo-like kinase (NLK) is involved in the suppression of Wnt target gene expression by phosphorylating TCF (in *C. elegans* called POP1), thereby inhibiting its DNA-binding affinity (12). Previous reports suggested that some members of TCF/LEFs are involved in the ubiquitin-proteasome pathway (13, 14, 15). For instance, TCF7L2 and LEF1 are ubiquitylated and degraded by an E3 ubiquitin ligase NARF, which is the abbreviation of NLK-associated RING finger protein (15). Despite the diverse and specific functions, TCF/LEFs are often expressed in overlapping patterns and the proteins display common structural features. Therefore, the regulators of the protein stability of all four TCF/LEFs may exist and need to be explored further.

The von Hippel-Lindau protein (pVHL) is the substrate recognition component of an E3 ubiquitin ligase complex (16). Inactivation of the *VHL* gene occurs in most hereditary von Hippel-Lindau disease and sporadic clear-cell renal carcinomas (16, 17). Well-documented canonical targets of pVHL are the α subunits of hypoxia-inducible factors (HIF-α) (18, 19). In addition, accumulated evidence has indicated that pVHL also participates in the regulation of Wnt/β-catenin signaling. A previous study reported that pVHL regulates Wnt/β-catenin signaling by reducing β-catenin protein levels through Jade-1 (20, 21). Additionally, pVHL binds to DVL and ubiquitinates it, facilitating DVL degradation via the autophagy–lysosome pathway (22). pVHL binding protein 1 (VBP1) was identified as a binding partner of pVHL by a yeast two-hybrid assay (23). Several studies have suggested that VBP1 exerts its function via pVHL. For example, VBP1 mediated the degradation of HIV-1 integrase together with pVHL (24). Specifically, VBP1 bound to HIV-1 integrase and promoted its binding to the E3 ubiquitin ligase complex composed of pVHL and elongin B/C, resulting in the ubiquitination and proteasomal degradation of HIV-1 integrase (24). Additionally, VBP1 bound to a mismatch repair protein family member, hMSH4, and promoted its degradation by pVHL-mediated ubiquitin-proteasome and p97-dependent autophagy pathways (25). Furthermore, *Drosophila* VBP1 homologue Mgr regulated tubulin degradation by cooperating with pVHL (26). A recent study highlighted that VBP1 also stabilized pVHL by inhibiting the ubiquitination of pVHL and then facilitated pVHL-mediated degradation of HIF-1α (27). Given that pVHL regulates Wnt/β-catenin signaling at multiple levels and VBP1 functions as a pVHL binding protein, however, whether VBP1 also regulates Wnt/β-catenin signaling remains unknown.

In the present study, we investigated the role of VBP1 in regulating Wnt/β-catenin signaling both *in vitro* and *in vivo*. Unexpectedly, our results demonstrated that either overexpression or knockdown of VBP1 decreased Wnt/β-catenin signaling activity *in vitro* and *in vivo*. We revealed that VBP1 directly binds to all four TCF/LEF family members and to pVHL. Intriguingly, either overexpression or knockdown of VBP1 in-creased the interaction between TCF/LEFs and pVHL, which then promoted TCF/LEFs protein degradation via the proteasomal pathway. Using a pVHL deficient cell line, we uncovered that VBP1 regulated TCF/LEF protein stability via pVHL. Together, our study provides insights into the mechanism of protein stability regulation of all TCF/LEF family members by VBP1.

## Results

### Overexpression of VBP1 inhibited Wnt/β-catenin signaling in vitro and in vivo

**Figure 1:**
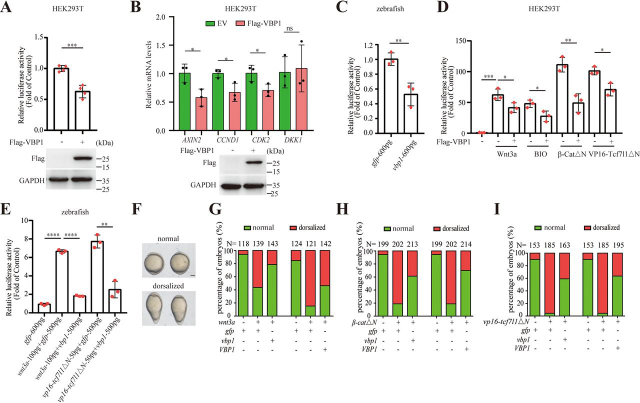
**Overexpression of VBP1 inhibits Wnt/β-catenin signaling.***A*, TOPFlash luciferase assays in HEK293T cells with 500-ng FLAG-tagged VBP1 plasmid transfection. Expression of FLAG-VBP1 was confirmed by Western blotting. Data are from four independent experiments with individual data points shown. *B*, relative mRNA levels of indicated Wnt target genes in control or VBP1-overexpressing HEK293T cells were analyzed by qRT-PCR. Data are from three independent experiments with individual data points shown. *C*, TOPFlash luciferase assays in zebrafish embryos with 600-pg *VBP1* mRNA injection. Data are from three independent experiments with individual data points shown. *D*, TOPFlash luciferase assays in Wnt3a-, BIO-, β-CatΔN-, or VP16-Tcf7l1ΔN-treated HEK293T cells with VBP1 overexpression. Control or VBP1-overexpressing HEK293T cells were cotransfected with *Renilla* and TOPFlash plasmids. The activation of Wnt/β-catenin signal was induced by BIO (1 µm) treatment or by indicated plasmid DNAs (15 ng of Wnt3a, 30 ng of β-CatΔN, or 50 ng of VP16-Tcf7l1ΔN) transfection. Data are from three independent experiments with individual data points shown. *E*, TOPFlash luciferase assays in *wnt3a*- or *vp16-tcf7l1*Δ*N*-injected zebrafish embryos with *VBP1* overexpression. TOPFlash and *Renilla* plasmids were co-injected into embryos with *gfp* or *VBP1* mRNA. The activation of Wnt/β-catenin signal was induced by injecting 100 pg of *wnt3a* mRNA or 50 pg of *vp16-tcf7l1*Δ*N* mRNA. Data are from three independent experiments with individual data points shown. *F*, overexpression of *VBP1*/*vbp1* antagonized *wnt3a-*, β*-cat*Δ*N-*, or *vp16-tcf7l1*Δ*N-*induced dorsalized phenotype in zebrafish embryos. Lateral view of representative injected embryos at 5-somite stage was shown. *Scale bar*, 200 µm. *G*–*I*, quantitation of embryos displaying normal or dorsalized phenotype in *F*. Data are from three independent experiments, and the embryo number of each group is shown on the top. The values are mean ± S.D. Unpaired *t* test. *p < 0.05; **p < 0.01; ***p < 0.001; ****p < 0.0001; *ns*, nonsignificant.

Because pVHL regulates the Wnt/β-catenin pathway at multiple levels and VBP1 serves as a binding protein to cooperate with pVHL in many biological processes (24, 25, 26), we assume that VBP1 may participate in the regulation of the Wnt/β-catenin pathway. To test this possibility, we examined the effect of VBP1 on Wnt/β-catenin signaling both *in vitro* and *in vivo*. We first used the TOPFlash reporter, a well-established Wnt/β-catenin signal-responsive reporter, which contains tandem repeats of the TCF/LEF response element, to examine the role of VBP1 in the Wnt/β-catenin pathway in cultured cell lines. Transfection of HEK293T cells with a plasmid encoding the FLAG-tagged VBP1 protein significantly decreased basal Wnt/β-catenin reporter activity (Fig. 1*A*). In addition, overexpression of VBP1 decreased the expression level of several Wnt/β-catenin direct target genes including *AXIN2*, *CCND1*, and *CDK2* in HEK293T cells (Fig. 1*B*). Interestingly, the expression of *DKK1*, which is a repressor of the Wnt/β-catenin pathway was not changed by VBP1 overexpression. To determine whether Wnt/β-catenin signaling was affected by VBP1 *in vivo*, the capped mRNA of *VBP1* was generated and injected into 1–2-cell stage zebrafish embryos, and then the Wnt/β-catenin reporter activity was measured at 6 hpf (hours postfertilization). As shown in Fig. 1*C*, forced expression of *VBP1* mRNA inhibited endogenous Wnt/β-catenin activity in zebrafish embryos. Taken together, these results suggested that overexpression of VBP1 inhibited endogenous Wnt/β-catenin signaling activity *in vitro* and *in vivo*.

To further confirm whether VBP1 indeed regulated the Wnt/β-catenin signaling pathway, we investigated the genetic interaction between VBP1 and the core components of the Wnt/β-catenin signaling pathway in HEK293T cells and zebrafish embryos. Induction of Wnt/β-catenin signals by Wnt3a, 6-Bromoindirubin-3′-oxime (BIO) (GSK3β inhibitor) (28, 29), constitutively activated β-catenin (β-CatΔN), or constitutively activated Tcf7l1 (VP16-Tcf7l1ΔN, a β-catenin–independent VP16-Tcf7l1 fusion protein that lacks the β-catenin-binding site) in HEK293T cells resulted in dramatically increased Wnt/β-catenin reporter activity (Fig. 1*D*). Overexpression of VBP1 inhibited Wnt/β-catenin reporter activity induced by Wnt3a, BIO, β-CatΔN, and VP16-Tcf7l1ΔN in HEK293T cells (Fig. 1*D*). Similarly, overexpression of VBP1 inhibited Wnt/β-catenin reporter activity induced by Wnt3a and VP16-Tcf7l1ΔN in zebrafish embryos (Fig. 1*E*). Taken together, the above results implied that VBP1 is likely to inhibit Wnt/β-catenin activity at the TCF/LEF level. To further confirm the inhibitory effect of VBP1 on Wnt/β-catenin signaling *in vivo*, we performed epistatic analysis between VBP1 and the core components of the Wnt/β-catenin signaling pathway on the formation of the dorsoventral axis in zebrafish embryos. Because the VBP1/Vbp1 amino acid sequence is highly conserved in vertebrates from fish to mammals (Fig. S1A), we co-injected human *VBP1* or zebrafish *vbp1* mRNA and mRNA for *wnt3a*, β*-cat*Δ*N*, or *vp16-tcf7l1*Δ*N* into zebrafish embryos. Consistent with a previous report (30), injection of *wnt3a*, β*-cat*Δ*N*, or *vp16-tcf7l1*Δ*N* mRNA into zebrafish embryos resulted in dorsalized phenotypes at 12.5 hpf, which were neutralized by co-injection with either human *VBP1* or zebrafish *vbp1* mRNA, suggesting that the Wnt inhibitory action of VBP1/Vbp1 is evolutionarily conserved and acts at the TCF/LEF level (Fig. 1, *F*–*I*). Collectively, these results suggested that overexpression of VBP1/Vbp1 inhibits Wnt/β-catenin signaling at TCF/LEF level *in vitro* and *in vivo*.

### Knockdown of VBP1 reduced Wnt/β-catenin signaling and TCF-dependent transcriptional activation

**Figure 2:**
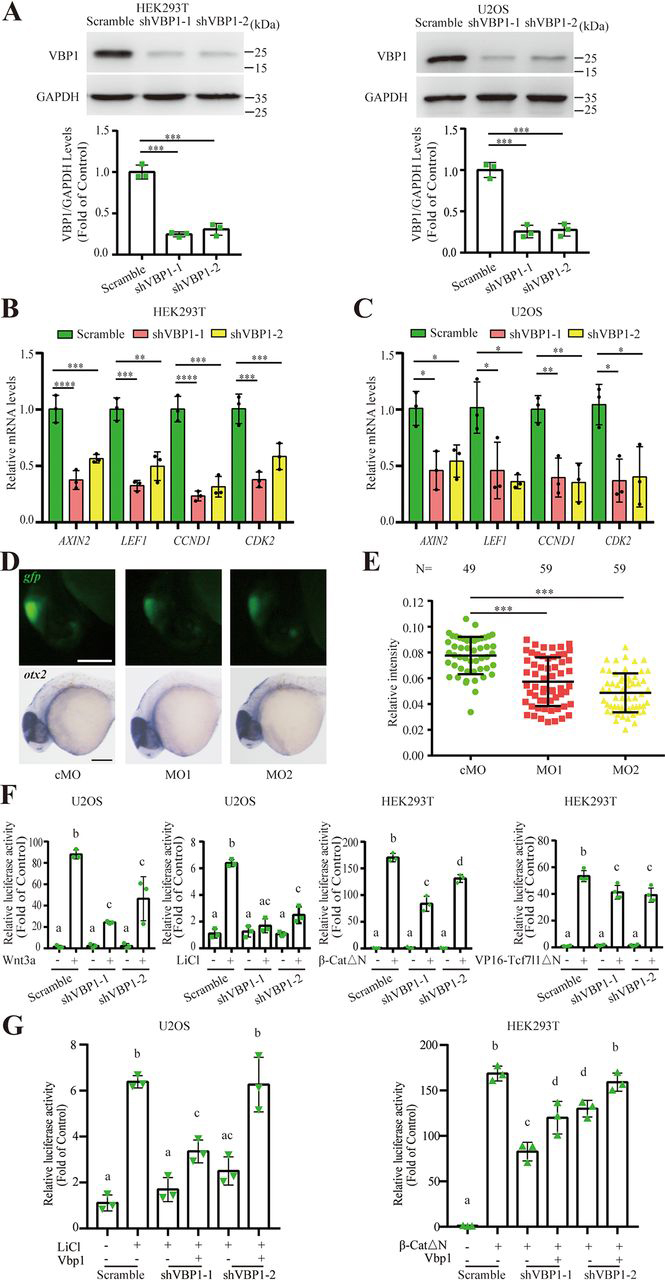
**VBP1 is required for Wnt/β-catenin signaling activity.***A*, VBP1 protein levels in stable shRNA-treated HEK293T and U2OS cells. Densitometric analysis was from three independent experiments with individual data points shown. *B* and *C*, the transcriptional levels of indicated Wnt target genes in control or VBP1-depleted HEK293T (*B*) and U2OS (*C*) cells were analyzed by qRT-PCR. Data are from three independent experiments with individual data points shown. *D*, representative images of injected embryos. GFP fluorescence was detected at the midbrain in TOPdGFP transgenic embryos at 24 hpf. The pattern of *otx2* expression did not change in the corresponding injected embryos. *Scale bar*, 200 µm. *E*, quantification of green fluorescence in (*D*) as relative fluorescence intensity. Image J software was used to measure the fluorescence intensity. Data are from three independent experiments, and the embryo number of each group is shown on the top. *F*, Wnt/β-catenin activity in Wnt3a-, LiCl-, β-CatΔN-, and VP16-Tcf7l1ΔN-treated cells with *VBP1* knockdown. TOPFlash plasmid was cotransfected with *Renilla* plasmid into control or *VBP1*-knockdown cells. Wnt activity was induced by LiCl (30 mm) or indicated plasmid DNAs (10 ng of Wnt3a, 15 ng of β-CatΔN, or 30 ng of VP16-Tcf7l1ΔN). Data are from three independent experiments with individual data points shown. *G*, TOPFlash luciferase assays in shRNA-expressed U2OS (*left*) or HEK293T cells (*right*). Wnt/β-catenin activity was rescued by a shRNA-resistant zebrafish Vbp1. The activation of Wnt/β-catenin signal was induced by LiCl treatment (*left*) or transfection with β-CatΔN (*right*) plasmid DNA. Data are from three independent experiments with individual data points shown. The values are mean ± S.D. Unpaired *t* test. *p < 0.05; **p < 0.01; ***p < 0.001; ****p < 0.0001. One-way analysis of variance followed by Tukey's post-test. Groups labeled with different letters are significantly different from each other.

To delineate the role of endogenous VBP1 in Wnt/β-catenin signaling, HEK293T and U2OS cell lines stably expressing VBP1 shRNA were established. The knockdown efficiency of VBP1 was confirmed by Western blotting analysis, and each shRNA greatly reduced the VBP1 protein levels (Fig. 2*A*). Using these cell lines, we tested whether *VBP1* knockdown affected the expression of Wnt/β-catenin target genes. As shown in Fig. 2*B*, knockdown of *VBP1* significantly reduced the expression level of Wnt/β-catenin target genes, including *AXIN2*, *LEF1*, *CCND1*, and *CDK2* in HEK293T cells. Similar results were observed in U2OS cells (Fig. 2*C*). These results suggested that knockdown of *VBP1* reduced endogenous Wnt/β-catenin signaling *in vitro*. We next determined the effect of Vbp1 knockdown on the Wnt/β-catenin signaling activity *in vivo* using a transgenic zebrafish Wnt/β-catenin reporter line, also known as the TOPdGFP reporter line. GFP expression in the zebrafish TOPdGFP reporter line was controlled by a promoter with multiple TCF/LEF response elements, which allowed the determination of Wnt/β-catenin activity and the identification of responsive cells through the expression of GFP protein (31). To ascertain the cell or tissue that we should observe in zebrafish embryos, we first investigated the spatiotemporal expression pattern of *vbp1* during embryogenesis using RT-PCR and whole-mount *in situ* hybridization. We found that the *vbp1* transcript was maternally deposited and ubiquitously expressed before 24 hpf (Fig. S1, B and C); however, its expression was enriched in the midbrain region at 24 hpf (Fig. S1C). Coincidentally, the midbrain region at 24 hpf was also rich in the strong fluorescence signal in the Wnt/β-catenin reporter line (31), suggesting that this region is suitable for observing the effect on the Wnt/β-catenin reporter after depletion of Vbp1. Knockdown of Vbp1 was performed using two nonoverlapping translation-blocking morpholinos (MOs) because of translation-blocking MO inhibited translation of maternally deposited mRNA. The effectiveness of MOs (MO1 and MO2) was confirmed by blocking the translation of a *vbp1* 5′-UTR-GFP reporter plasmid, and both MOs significantly blocked the translation of GFP mRNA in zebrafish embryos (Fig. S1D). Subsequently, control MO (8 ng), MO1 (4 ng), or MO2 (8 ng) was injected into TOPdGFP transgenic embryos at the 1–2-cell stage, respectively. In control MO–injected embryos, a strong green fluorescence signal, indicative of Wnt/β-catenin activity, was observed in the midbrain region at 24 hpf (Fig. 2*D*). In contrast, the GFP intensity was dramatically reduced in the Vbp1 MO-injected embryos (Fig. 2, *D* and *E*). The decrease of fluorescence intensity was not because of morphological changes in the midbrain, which were indicated by the normal expression of midbrain marker *otx2* (Fig. 2*D*). These results suggested that depletion of Vbp1 in zebrafish embryos decreased the endogenous Wnt/β-catenin activity *in vivo*. Collectively, depletion of VBP1/Vbp1 reduced endogenous Wnt/β-catenin signaling both *in vitro* and *in vivo*.

We next investigated at which level VBP1 knockdown reduced Wnt/β-catenin activity. Again, Wnt/β-catenin activity was induced by Wnt3a, LiCl (GSK3β inhibitor) (32), β-CatΔN, and VP16-Tcf7l1ΔN, at multiple levels (Fig. 2*F*). Knockdown of VBP1 in cultured cells dramatically reduced the Wnt/β-catenin activity induced by these molecules (Fig. 2*F*). These results implied that depletion of VBP1 reduced Wnt/β-catenin signaling at the TCF/LEF transcriptional level. We further tested whether the effect of knockdown of VBP1/Vbp1 on Wnt/β-catenin signaling is specific. Because the effect of VBP1/Vbp1 on Wnt/β-catenin signaling is evolutionarily conserved between zebrafish and humans, we introduced zebrafish Vbp1 into VBP1-depleted human cells to test whether zebrafish Vbp1 could rescue the reduced Wnt/β-catenin activity in cultured human cells. As expected, expression of shRNA-resistant zebrafish Vbp1 significantly rescued the reduced Wnt/β-catenin activity by *VBP1* knockdown in the background of LiCl treatment and β-CatΔN overexpression (Fig. 2*G*). These results indicated that the reduction of Wnt signaling by *VBP1* knockdown was specific and that the effect of knockdown of VBP1/Vbp1 on Wnt/β-catenin signaling was evolutionarily conserved. Taken together, our results demonstrated that depletion of VBP1/Vbp1 attenuated Wnt/β-catenin signaling at the TCF/LEF transcriptional level.

### Overexpression or knockdown of VBP1 promoted degradation of TCF/LEFs

**Figure 3:**
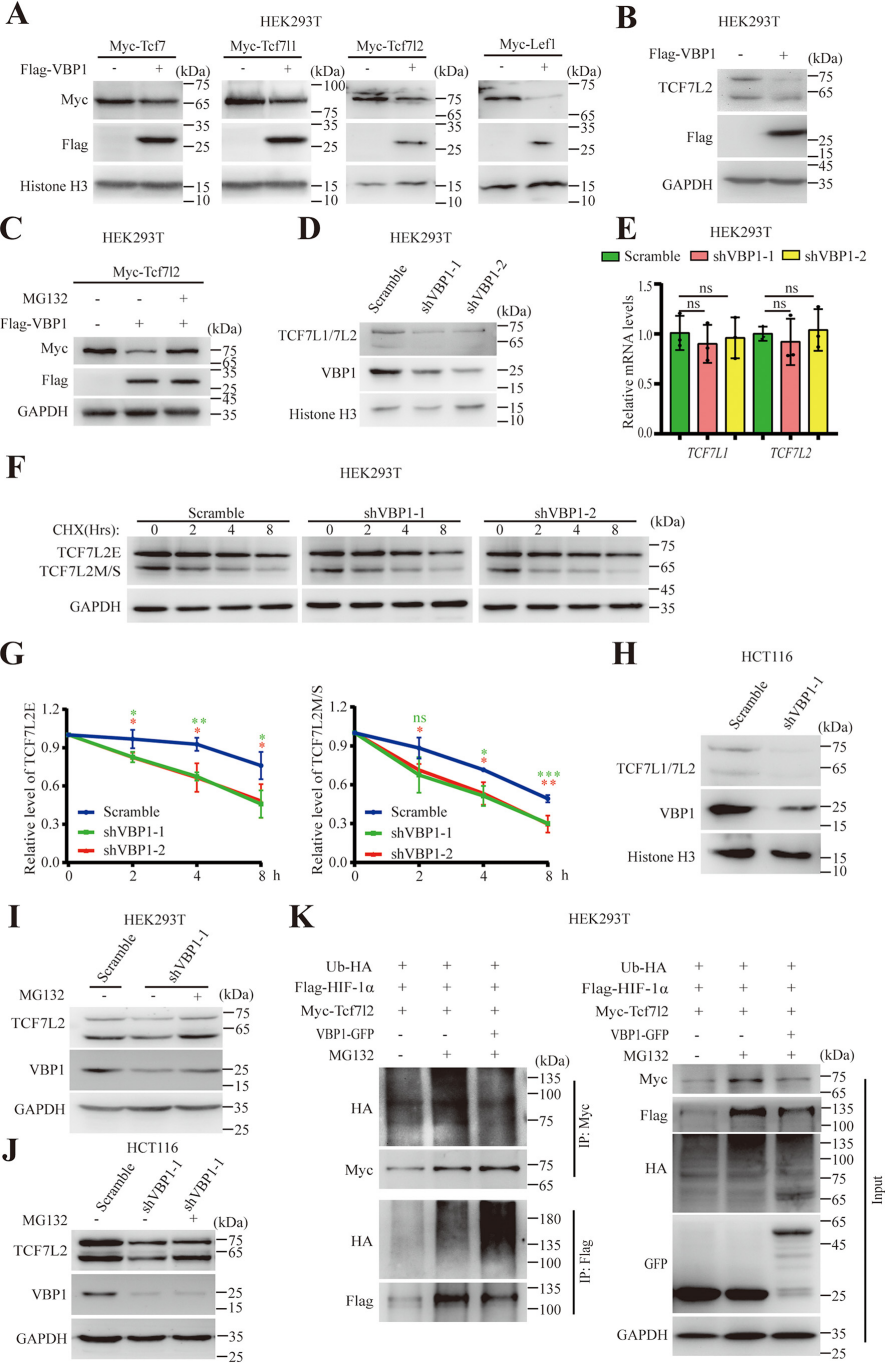
**Overexpression or knockdown of VBP1 promotes protein degradation of TCF/LEFs.***A*, exogenous Tcf/Lef protein levels in control or VBP1-overexpressing HEK293T cells. Similar results were obtained from four experiments. *B*, endogenous TCF7L2 protein level in control or VBP1-overexpressing HEK293T cells. Similar results were obtained from three experiments. *C*, changes in exogenous TCF7L2 protein level in VBP1-overexpressing HEK293T cells treated with MG132. Cells transfected with indicated plasmid DNA and treated with or without 10 µm MG132 for 8 h. Similar results were obtained from three experiments. *D*, TCF7L1 and TCF7L2 protein levels in control or *VBP1*-knockdown HEK293T cells. Similar results were obtained from three experiments. *E*, qRT-PCR analysis of transcriptional levels of *TCF7L1* and *TCF7L2* in control and *VBP1*-knockdown HEK293T cells. Data are from three independent experiments with individual data points shown. *F*, representative image of TCF7L2 protein levels in control or *VBP1*-knockdown HEK293T cells treated with CHX in a time series. *G*, quantitation of relative protein levels from the two isoforms of TCF7L2, TCF7L2E (*left panel*) and TCF7L2M/S (*right panel*), in (*F*), respectively. The band intensity was quantified using ImageJ software. Data are from three independent experiments. *H*, TCF7L1 and TCF7L2 protein levels in control or *VBP1*-knockdown HCT116 cells. Similar results were obtained from three experiments. *I*, changes in TCF7L2 protein levels in *VBP1*-knockdown HEK293T cells treated with MG132. VBP1-depleted HEK293T cells treated with or without 10 µm MG132 for 8 h. Similar results were obtained from three experiments. *J*, changes in TCF7L2 protein levels in *VBP1*-knockdown HCT116 cells treated with MG132. *VBP1*-knockdown HCT116 cells treated with or without 10 µm MG132 for 8 h. Similar results were obtained from three experiments. *K*, ubiquitylation assays in HEK293T cells transfected with indicated plasmids. HIF-1α was used as a positive control of ubiquitylation promoted by VBP1. Similar results were obtained from three experiments. The values are mean ± S.D. Unpaired *t* test. *p < 0.05; **p < 0.01; ***p < 0.001; *ns*, nonsignificant.

Given that either overexpression or knockdown of VBP1 attenuated Wnt/β-catenin signaling at the TCF/LEF level and VBP1 is a binding protein of pVHL, which is a subunit of an E3 ubiquitin ligase complex, we speculated that VBP1 might regulate TCF/LEF protein stability. To this end, we co-overexpressed VBP1 and TCF/LEFs in HEK293T cells. When cells were cotransfected with plasmids containing Myc-tagged TCF/LEFs and FLAG-tagged VBP1, exogenous TCF/LEF protein levels were reduced (Fig. 3*A*). In addition, overexpression of VBP1 reduced the endogenous TCF7L2 protein levels in HEK293T cells (Fig. 3*B*). This effect was restored after the addition of the proteasome inhibitor MG132 (Fig. 3*C*), indicating that TCF7L2 degradation promoted by overexpressed VBP1 depended on the proteasomal pathway.

We next examined the effect of *VBP1* knockdown on TCF/LEF protein levels. Knockdown of VBP1 decreased the protein levels of endogenous TCF7L1 and TCF7L2 in HEK293T cells (Fig. 3*D*). In contrast, the expression of *TCF7L1* and *TCF7L2* mRNA was not changed upon the depletion of VBP1 when measured by qRT-PCR analysis (Fig. 3*E*). These results implied that TCF7L1 and TCF7L2 protein levels were regulated by VBP1 through a posttranslational mechanism. To determine whether knockdown of VBP1 accelerated degradation of TCF7L2, we measured TCF7L2 *t*_1/2_ in HEK293T cells treated with cycloheximide (CHX), a protein synthesis inhibitor. The *t*_1/2_ of TCF7L2 was significantly shortened when VBP1 was knocked down (Fig. 3, *F* and *G*). Because the HEK293T cell line was regarded as a Wnt-off cell line, we want to determine whether knockdown of VBP1 regulates TCF/LEF protein stability in Wnt-activated colon cancer cells. Knockdown of VBP1 also decreased TCF7L1 and TCF7L2 protein levels in Wnt-activated HCT116 colon cancer cells, which harbor an oncogenic mutation in β-catenin (Fig. 3*H*). These data suggested that knockdown of VBP1 reduced TCF/LEF protein stability in both active and inactive Wnt/β-catenin signaling status. Furthermore, the reduction of TCF7L2 protein levels by *VBP1* depletion was restored by MG132 treatment in both HEK293T and HCT116 cells (Fig. 3, *I* and *J*), indicating that VBP1 depletion–mediated TCF7L2 degradation was also dependent on the proteasomal pathway. Protein degradation by the proteasomal pathway may be ubiquitin-dependent and ubiquitin-independent (33). To address the type of TCF/LEF protein degradation, we examined the ubiquitylation of Tcf7l2 under the VBP1-overexpressed condition. Myc-Tcf7l2 and FLAG–HIF-1α were cotransfected with VBP1 simultaneously into HEK293T cells, and then ubiquitylation analysis was performed after immunoprecipitation. VBP1 induced a dramatic enhancement in the ubiquitylation of HIF-1α, as previously reported (27), but failed to induce the ubiquitylation of Tcf7l2. Considering that the reduction of Tcf7l2 protein levels by overexpression or depletion of VBP1 was restored in addition of a proteasome inhibitor, our data suggested that Tcf7l2 likely underwent a ubiquitin-independent proteasomal pathway (Fig. 3*K*). Taken together, these results implied that *VBP1* depletion promoted degradation of the TCF7L2 in both basal and activated Wnt/β-catenin activity status via proteasomal pathway.

### VBP1 interacted with TCF/LEFs and promoted their degradation through pVHL

**Figure 4:**
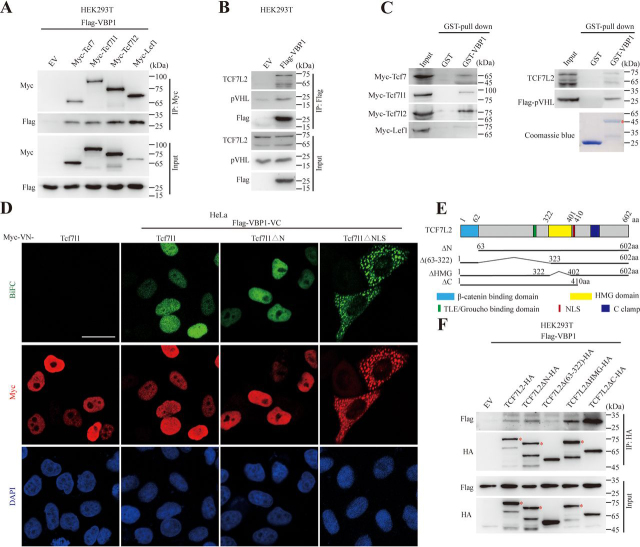
**VBP1 interacts with TCF/LEFs and pVHL.***A*, the interaction between exogenous TCF/LEFs and VBP1. HEK293T cells were cotransfected with FLAG-tagged VBP1 and Myc-Vector, Myc-Tcf7, Myc-Tcf7l1, Myc-Tcf7l2, or Myc-Lef1, respectively. After 48 h, the cell extracts were immunoprecipitated with an anti-Myc antibody. The immunoprecipitates and the inputs were analyzed by Western blotting with indicated antibody. Similar results were obtained from three experiments. *B*, the interaction among exogenous VBP1, endogenous TCF7L2, and pVHL. HEK293T cells were transfected with FLAG-tagged VBP1. After 48 h, the cell extracts were immunoprecipitated with an anti-FLAG antibody. The immunoprecipitates and the inputs were analyzed by Western blotting with indicated antibody. Similar results were obtained from three experiments. *C*, VBP1 bound directly to TCF/LEF. GST and GST-VBP1 proteins expressed by bacteria were incubated with GSH beads and the extracts from nontransfected HEK293T cells (for endogenous TCF7L2 detection) or HEK293T cells transfected with Myc-Tcf/Lefs or FLAG-pVHL. *Red asterisk* indicates the specific band of GST-VBP1. Similar results were obtained from three experiments. *D*, BiFC assays detected the binding of VBP1 to Tcf7l1/Tcf7l1ΔN/Tcf7l1ΔNLS in HeLa cells. HeLa cells were cotransfected with FLAG-VBP1-VC and Myc-VN-Tcf7l1, Myc-VN-Tcf7l1ΔN, or Myc-VN-Tcf7l1ΔNLS, respectively. After 24h, Venus in *green* indicates the interaction between VN-tagged and VC-tagged proteins. Tcf7l1/Tcf7l1ΔN/Tcf7l1ΔNLS was detected by an anti-Myc antibody (*red*). Nucleus was stained with 4′,6-diamidino-2-phenylindole (*blue*). *Scale bar*, 10 µm. Similar results were obtained from three experiments. *E* and *F*, mapping of the region in TCF7L2 responsible for the VBP1 interaction. Schematic diagram of human TCF7L2 protein domains is shown in (*E*). Various HA-tagged TCF7L2 deletion mutants were cotransfected with FLAG-tagged VBP1 in HEK293T cells and cell lysates were subjected to co-immunoprecipitation (*F*). *Red asterisk* indicates the specific band. Similar results were obtained from three experiments.

To understand the mechanism underlying the regulation of TCF/LEF protein stability by VBP1, we first tested the possible interaction between VBP1 and TCF/LEFs. The co-immunoprecipitation (Co-IP) assay detected VBP1 in the immunoprecipitates of four Myc-tagged TCF/LEF family members in HEK293T cells (Fig. 4*A*). Additionally, when FLAG-tagged VBP1 was overexpressed in HEK293T cells, it formed a complex with endogenous TCF7L2 and pVHL (Fig. 4*B*). Furthermore, purified GST–VBP1 protein pulled down all four Myc-tagged TCF/LEF members (Fig. 4*C*, *left panel*). Likewise, a direct protein-protein interaction among VBP1, endogenous TCF7L2, and FLAG-tagged pVHL was further confirmed by the GST pulldown assay (Fig. 4*C*, *right panel*). These results indicated that VBP1 directly interacted with TCF/LEF and pVHL in HEK293T cells. We next assessed whether VBP1 and Tcf7l1 interact with each other in living HeLa cells using a previously established bimolecular fluorescence complementation (BiFC) assay (34). As shown in Fig. 4*D*, the green BiFC signals indicated that VBP1 interacted with both Tcf7l1 and Tcf7l1ΔN in the nucleus, whereas VBP1 bound to Tcf7l1ΔNLS (lacking the nuclear localization signal) in the cytoplasm, suggesting that VBP1 and Tcf7l1 did interact with each other in living cells. Subsequently, we generated a series of TCF7L2 domain-deleted mutants to map the region of TCF7L2 involved in the interaction with VBP1 (Fig. 4*E*). By Co-IP assay, we found that the TLE/Groucho binding region–deleted mutant of TCF7L2 exhibited rather weak interactions with VBP1 compared with WT TCF7L2, high mobility group box–deleted, or C-terminal region–deleted mutant (Fig. 4*F*). This suggests that the TLE/Groucho binding region in TCF7L2 may play an important role for its interaction with VBP1.

**Figure 5:**
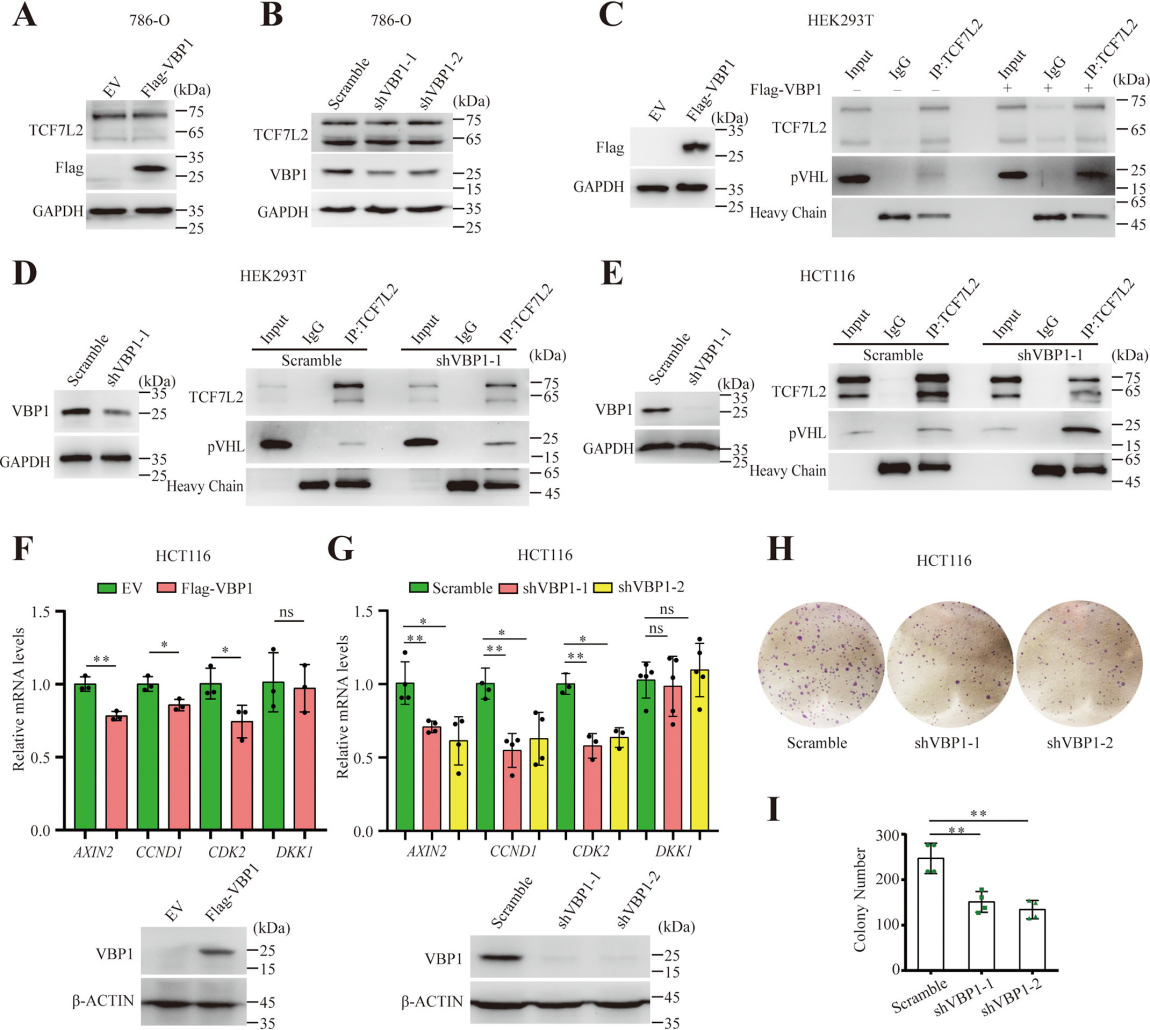
**VBP1 regulates TCF/LEFs protein stability through pVHL.***A*, TCF7L2 protein levels in control or VBP1-overexpressing 786-O cells. Similar results were obtained from three experiments. *B*, TCF7L2 protein levels in control or *VBP1*-knockdown 786-O cells. Similar results were obtained from three experiments. *C*, the association between endogenous TCF7L2 and pVHL in control or VBP1-overexpressing cells. HEK293T cells were transfected with FLAG-tagged VBP1. After 48 h, the cell extracts were immunoprecipitated with an anti-TCF7L2 antibody. The immunoprecipitates and the inputs were analyzed by Western blotting with indicated antibody. *D* and *E*, the association between endogenous TCF7L2 and pVHL in control or *VBP1*-knockdown HEK293T (*D*) and HCT116 (*E*) cells. Cell extracts were immunoprecipitated with an anti-TCF7L2 antibody. The immunoprecipitates and the inputs were analyzed by Western blotting with indicated antibody. Similar results were obtained from three experiments. *F* and *G*, the transcriptional levels of indicated Wnt target genes in VBP1-overexpressing (*F*) or -depleted (*G*) HCT116 cells were analyzed by qRT-PCR. FLAG-VBP1 or endogenous VBP1 protein levels in VBP1-overexpressing or -depleted HCT116 cells were analyzed by Western blotting. Data are from 3–5 independent experiments with individual data points shown. *H*, representative images of colony formation assay were shown. *I*, quantification of the colony number shown in (*H*). Data are from four independent experiments with individual data points shown. Values are means ± S.D. Unpaired *t* test, two-tailed. *p < 0.05; **p < 0.01; *ns*, nonsignificant.

Of note, pVHL was also present in the complex containing VBP1 and TCF/LEF (Fig. 4*B*). In addition, VBP1, as a pVHL binding protein, participates and regulates pVHL-related biological processes. We speculated that VBP1-mediated regulation of TCF/LEF stability was related to pVHL function. We, therefore, overexpressed or knocked down VBP1 protein in *VHL*-deficient clear-cell renal carcinoma 786-O cells. As expected, TCF7L2 protein levels were not changed in 786-O cells with either overexpression or knockdown of VBP1 (Fig. 5, *A* and *B*). These data suggested that pVHL is required for VBP1 to regulate the stability of TCF/LEFs. We next performed a series of Co-IP analyses to detect the association between endogenous TCF7L2 and pVHL with VBP1 overexpression or knockdown in different cell lines of distinct Wnt/β-catenin activity. We observed that the association between endogenous TCF7L2 and pVHL increased under the condition of either VBP1 overexpression or knockdown in HEK293T cells (Fig. 5, *C* and *D*). Similarly, knockdown of VBP1 in Wnt-activated HCT116 colon cancer cells also increased the interaction between TCF7L2 and pVHL (Fig. 5*E*). These results indicated that the presence of pVHL is required for VBP1 to regulate the stability of TCF/LEFs and that alteration of VBP1 protein levels increases the association between TCF/LEF and pVHL, which may promote the degradation of TCF/LEF proteins.

Because colorectal cancers often display overactivated Wnt/β-catenin signaling and VBP1 regulates the protein stability of TCF/LEFs in HCT116 colon cancer cells, we next determined whether VBP1 has a physiological role in colorectal cancer cells. The alterations of Wnt/β-catenin target genes were measured in HCT116 cells after overexpression or knockdown of VBP1. Similar with the effect of VBP1 in HEK293T cells, either overexpression or knockdown of VBP1 in HCT116 cells reduced the expression of Wnt/β-catenin target genes, including *AXIN2*, *CCND1*, and *CDK2*, but did not reduce the expression of Wnt/β-catenin signaling pathway negative feedback gene, *DKK1* (Fig. 5, *F* and *G*). These results suggested that VBP1 might have physiological roles in colorectal cancers. It is well-known that activation of Wnt/β-catenin signaling promotes proliferation of colorectal cancer cell. It is interesting to know whether alternation of VBP1 decreases the proliferation of colorectal cancers. We, therefore, examined the effect of VBP1 knockdown on proliferation of HCT116 cells using a colony formation assay. Our results indicated that depletion of VBP1 inhibited the proliferation of HCT116 cells, which was highlighted by the significant reduction in the number of colonies (Fig. 5, *H* and *I*). These results suggest that VBP1 plays a physiological role in the cell proliferation of colorectal cancers.

## Discussion

In the present study, we show that VBP1 plays an important role in regulating the Wnt/β-catenin signaling pathway, which is known as an important signaling pathway that controls developmental processes in the embryo and regulates adult homeostasis and stem cell self-renewal (35). Transcription factors TCF/LEFs and their cofactor β-catenin represent the key components of the canonical Wnt pathway (36). However, compared with our knowledge on the regulation of β-catenin, posttranslational regulation of TCF/LEF proteins is poorly understood. Previous studies show that TCF family members LEF1, TCF7L2, and TCF7L1 are phosphorylated by HIPK2, leading to the dissociation of LEF1, TCF7L2, and TCF7L1 from their target promoter (37, 38). NLK inhibited the Wnt/β-catenin pathway via TCF7L2 and LEF1 phosphorylation, thus dissociating them from their target DNAs (12, 39, 40). A recent study showed that NARF was involved in the ubiquitylation of TCF7L2 and LEF1 as a RING finger-type E3 ubiquitin ligase (15). Here we found that VBP1 binds to all four TCF/LEF family members, and we also showed that either lower or excessive protein level of VBP1 facilitates the association between pVHL and TCF/LEFs and then promotes the degradation of TCF/LEFs via pVHL. Because it is possible that Wnt/β-catenin signals also regulate gene expression by dynamically controlling the concentration of TCF/LEFs, these findings provide evidence that VBP1 and pVHL are involved in regulation of protein levels of TCF/LEFs, and our results suggested that the stability of TCF/LEFs controlled by VBP1 and pVHL is critical for the regulation of Wnt/β-catenin signaling *in vitro* and *in vivo*.

We observed that overexpression of VBP1 decreased the expression of Wnt target genes and Wnt/β-catenin reporter activity *in vivo* and *in vitro*. Likewise, VBP1/Vbp1 antagonized the Wnt/β-catenin–induced dorsalizing activity in zebrafish embryos. Surprisingly, depletion of VBP1 also attenuated Wnt/β-catenin activity *in vivo* and *in vitro*. Furthermore, we demonstrated that VBP1 regulated the Wnt/β-catenin signaling pathway at the TCF/LEF-dependent transcriptional level by genetic interaction analysis. These data presented here raised an interesting question regarding lower or excessive VBP1 reducing Wnt/β-catenin signaling activity. To answer this question, we showed that either overexpression or knockdown of VBP1 reduced the stability of TCF/LEFs, which might have resulted from increased association between pVHL and TCF/LEFs when VBP1 was overexpressed or knocked down. Consequently, this reduced the activity of Wnt/β-catenin signaling. Similarly, a recent study revealed that either overexpression or knockdown of SRPK1 decrease the association between Akt and PHLPP1 and then interfere with PHLPP-mediated dephosphorylation of Akt, resulting in the promotion of cancer (41). This observation indicated that VBP1 may not be the only example that lower or excessive protein levels resulted in the same biological effect. Our findings suggest that the protein levels of VBP1 are tightly correlated with the stability of TCF/LEFs and reveal an unusual regulatory mechanism of the well-studied Wnt/β-catenin signaling pathway.

There is no evidence that VBP1 has any enzymatic activity to promote direct protein degradation. VBP1 was identified as a binding protein of pVHL, which is a component of an E3 ubiquitin ligase complex (42). VBP1 has been shown to cooperate with pVHL in regulating protein stability. For example, VBP1 regulated tubulin degradation together with pVHL (26). A similar partnership between VBP1 and pVHL was also reported. In brief, VBP1 mediated the interaction between HIV-1 integrase and pVHL, promoting integrase degradation (24). VBP1 also facilitated hMSH4 destruction partly through the pVHL-mediated proteasomal pathway (25). Based on these results, we postulated that VBP1 regulated TCF/LEF protein stability through pVHL. Indeed, our data demonstrated that VBP1 had no apparent effects on TCF/LEF protein stability in *VHL*-deficient 786-O cells. Moreover, our results showed that VBP1 directly interacted with TCF and pVHL. These observations suggested the possibility of VBP1 forming a complex with TCF and pVHL. Moreover, a recent study showed that VBP1 stabilizes pVHL by inhibiting the ubiquitination of pVHL and then facilitates pVHL-mediated ubiquitination of HIF-1α (27). A previous study shows that VBP1 localizes to the nucleus when it is co-overexpressed with pVHL (23). Based on these results, we suggested the following model of knockdown or overexpression of VBP1 promoting the degradation of TCF/LEFs through pVHL: at the basal level, VBP1 binds and protects TCF/LEFs from degradation. When VBP1 levels were lower in the cell and the association of pVHL with TCF/LEFs increased, proteasomal degradation of TCF/LEFs via pVHL occurred. In contrast, excessive VBP1 may stabilize pVHL and promote its translocation into the nucleus, also resulting in the increased interaction between pVHL and TCF/LEFs, and in turn promoted the degradation of TCF/LEFs. This model is based on the different binding ability of VBP1 and VHL with TCF/LEFs, which needs to be elucidated. This raises the potential of the coupled mechanism of the expression levels of VBP1, pVHL, and TCF/LEF, and the possibility needs to be systematically investigated in animal models in the future. Unexpectedly, VBP1 did not induce enhancement in the ubiquitylation of Tcf7l2 but increased the ubiquitylation levels of HIF-1α simultaneously. However, the addition of the proteasome inhibitor MG132 restored the protein level of TCF7L2 irrespective of VBP1 being overexpressed or depleted. This means that VBP1-mediated TCF/LEF degradation via pVHL likely undergoes the ubiquitin-independent proteasomal pathway. The exact molecular mechanism remains to be elucidated in the future.

It should be mentioned that pVHL regulates Wnt/β-catenin signaling by reducing β-catenin levels and promoting DVL degradation (21, 22). VBP1 might also regulate these components. Therefore, we cannot exclude the possibility that the output of Wnt/β-catenin activity might be compromised when VBP1 is overexpressed or knocked down.

In conclusion, our study demonstrated that VBP1 controlled the stability of all four TCF/LEFs, the important Wnt/β-catenin signaling effectors, by cooperating with pVHL. Thus, these results expanded our current knowledge on the regulation of TCF/LEF stability and modulation of the Wnt/β-catenin signaling pathway. However, further studies are still required to elucidate the precise mechanism by which pVHL and VBP1 regulate TCF/LEF stability. The TCF/LEF family in vertebrates contains four members, which are expressed in distinct but broadly overlapping patterns. Mutants of each appear as distinct phenotypes (43). In addition, dysregulation of Wnt/β-catenin signaling links various diseases. Further study on the relationship among VBP1, pVHL, TCF/LEF, and Wnt/β-catenin signaling in developmental process and diseases could be performed in the future.

## Experimental procedures

### Antibodies and reagents

Primary antibodies against Myc (sc-40), β-catenin (sc-7199), VBP1 (sc-390465), and pVHL (sc-135657) were purchased from Santa Cruz Biotechnology. Antibodies against TCF7L2 (2569), GFP (2555), and HA (3724) were obtained from Cell Signaling Technology. The antibody against TCF7L1/L2 (ab12065) was provided by Abcam. The antibody against GAPDH (D110016) was purchased from BBI Life Sciences (Crumlin, UK). The antibody against histone H3 (P30266) was obtained from Abmart. The FLAG M2 antibody (F1804) was provided by Sigma-Aldrich. The antibody against β-actin (abs137975) was obtained from Absin Bioscience. Small molecules CHX (66-81-9) and BIO (667463-62-9) were purchased from Sigma-Aldrich. MG132 (HY-13259) was from MedChemExpress.

### Plasmids

The plasmids pCS2-6×Myc-Tcf7, pCS2-6×Myc-Tcf7l1, pCS2-6×Myc-Tcf7l2, pCS2-6×Myc-Lef1, pCDNA3-Myc-VN-Tcf7l1, pCDNA3-Myc-VN-Tcf7l1ΔN, and pCDNA3-Myc-VN-Tcf7l1ΔNLS were gifts from Dr. Wei Wu (Tsinghua University, China). The plasmid pGEX-2T (28-9546-53) was obtained from GE Healthcare. pCS2-FLAG-VBP1, pCS2-FLAG-VBP1-VC, pGEX-2T-VBP1, pCDNA3.1-TCF7L2-HA, and pCS2-VP16-Tcf7l1ΔN were generated by PCR subcloning. Briefly, the human VBP1 ORF was amplified by PCR from HEK293T cell cDNA using the following primers: F, 5′-CGCGGATCCGCGCCACCAT-GGCGGCCGTTAAGGACAG-3′; R, 5′-CCGGAATTCTTA-TGCTTTGTTCTTGGTAGAGTC-3′. The resultant product was subcloned into the pCS2-GFP, pCS2-FLAG, or pGEX-2T, respectively. pCS2-GFP and pCS2-FLAG were obtained by inserting GFP or FLAG tag into pCS2+ vector (a gift from Dr. Cunming Duan, University of Michigan, Ann Arbor). TCF7L2 was amplified by PCR from RD cell cDNA using the following primers: F, 5′-CGGGATCCGCCACCATGCCGCAGCTGAACGGCGGT-GGAG-3′; R, 5′-CGGAATTCCTATTCTAAAGACTTG-GTGACGAGCG-3′. The resultant product and mutant were subcloned into the pCDNA3.1-HA (128034, Addgene). Vp16 and dnTcf7l1 ORF were amplified by PCR and subcloned into pCS2+ vector. Primers are shown below: dnTcf7l1-F, 5′-CCGGAATTCGATGCAGGATGCAGCA-TTCTTCAAGGG-3′; dnTcf7l1-R, 5′-CGCGTCTAGATCAGTCACTGGATTTGGTCACC-3′; Vp16-F, 5′-CCGG-AATTCGCCGCCATGGCTCCAAAGAAGAAGCGT-3′; Vp16-R, 5′-GGCGAATTCCCACCGTACTCGTCAATTCCAAGGGCAT-3′. All plasmids were verified by sequencing.

### Zebrafish strains

WT (Tübingen) and TOPdGFP zebrafish strains were used in this study. The embryos were incubated in Embryo Medium at 28.5 °C. The embryos were strictly staged according to standard methods (44). All experimental protocols were approved by and conducted in accordance with the Ethical Committee of Experimental Animal Care, Ocean University of China.

### Cell lines and transfections

HEK293T, HeLa, HCT116, and U2OS cell lines were purchased from ATCC and cultured in DMEM (HyClone) supplemented with 10% FBS (Gibco) and 1% penicillin/streptomycin at 37 °C in 5% CO_2_. 786-O cells were maintained in RPMI medium (HyClone) with the same supplement mix. The cell lines were analyzed with short tandem repeat profiling by ShCellBank (Shanghai, China). The contamination by mycoplasmas in culture cells was tested by EZ-PCR Mycoplasmas Detection Kit (BI, Kibbutz Beit-Haemek, Israel) every three months. Transfections of cells with plasmids were performed using Lipofectamine 2000 (Invitrogen) following the manufacturer's instructions.

### Luciferase assays

For cell-based luciferase assays, 8 × 10^5^ cells were seeded in a 12-well plate and then transfected with TOPFlash reporter and specific expression constructs. Transfection efficiency was normalized by cotransfection with a *Renilla* reporter. The cells were lysed with 1× Passive Lysis Buffer (Promega). The *in vivo* luciferase assay was performed as reported previously (30, 45). In brief, 1-cell stage embryos were injected with 100 pg of TOPFlash DNA and 20 pg of *Renilla* plasmid DNA, or mRNA plus 100 pg of TOPFlash DNA and 20 pg of *Renilla* plasmid DNA and raised to the shield stage. Two independent groups of embryos (each with >20 embryos) were lysed in Passive Lysis Buffer. TOPFlash/*Renilla* luciferase assays were performed using the Dual-Luciferase Reporter Assay Kit (Promega) according to the manufacturer's instructions.

### MO, mRNA synthesis, and microinjection

Two antisense MOs (Gene Tools, Philomath, OR, USA) were designed for targeting the translation start site of zebrafish *vbp1*. The sequences were MO1 (5′-ATTCAAATGTAGTCCAT-CTGTCTTT-3′) and MO2 (5′-CAATGAGCAGGTGATGA-TTTCATCT-3′). The sequence of control MO was: 5′-CCT-CTTACCTCAGTTACAATTTATA-3′. The target sequence (−216 bp to 381 bp) was fused to GFP-coding sequences and subcloned into pCS2+ to generate a reporter for MO-knockdown efficiency test.

Capped mRNAs were synthesized using the mMESSAGE mMACHINE SP6 kit (Ambion, Austin, TX, USA) according to the manufacturer's instructions. All morpholinos and mRNAs were injected into the yolks of zebrafish embryos at the 1–2-cell stage and then embryos were raised to the indicated stages.

### Whole-mount in situ hybridization

Whole-mount *in situ* hybridization was performed as previously described (30, 45). The *vbp1* and *otx2* riboprobes were synthesized with T7 RNA polymerase (Promega). Stained embryos or live embryos were mounted in glycerol or 5% methylcellulose respectively, and images were taken using a Nikon SMZ1500 microscope.

### Quantitative RT-PCR

Total RNA was isolated using RNAiso plus reagent (Takara Bio), and 2.0 μg of total RNA was used as a template for reverse transcription. The cDNA was synthesized using the M-MLV reverse transcriptase (Promega). Quantitative PCR analyses were carried out with the iTaq SYBR GREEN Supermix and iCycler apparatus (Bio-Rad). Each sample was analyzed in duplicate, and qPCR primers were as follows: *GAPDH*, forward (F): 5′-TCGACAGTCAGCCGCATCTTCTT-3′ and reverse (R): 5′-GCGC-CCAATACGACCAAATCC-3; *LEF1*, F: 5′-CAGTCATCCCGAAGAGGAAG-3′ and R: 5′-AGGGCTCCTGAGAGGTT-TGT-3′; *AXIN2*, F: 5′-CTGGCTTTGGTGAACTGTTG-3′ and R: 5′-AGTTGCTCACAGCCAAGACA-3′; *CCND1*, F: 5′-CCATCCAGTGGAGGTTTGTC-3′ and R: 5′-AGCGTATCGTAGGAGTGGGA-3′; *CDK2*, F: 5′-CTCTTCCCCTCATCAAGAGCTA-3′ and R: 5′-ATTTGCAGCCCAGGAGGATT-3′; *DKK1*, F: 5′-GTGCAAATCTGTCTCGCCTG-3′ and R: 5′-CTGATGACCGGAGACAAACAG-3′; *TCF7L1*, F: 5′-GTCAACGAGTCGGAGAACCA-3′ and R: 5′-TCTCACTTCGGCGAAATAGTC-3′; *TCF7L2*, F: 5′- CCTCGGCAGAGAGGGATTTAGCTG-3′ and R: 5′-GAGCCCTCCATCTTGCCTCTTG-3′. Gene expression analysis was performed using the comparative ΔΔCT method with GAPDH normalization. Template-free negative controls were included in each experiment.

### Immunoblots and immunoprecipitation

Immunoblot and Co-IP were performed as described previously (46). In brief, 1 × 10^6^ cells were seeded in a 35-mm dish and the cells were harvested at 24 h posttransfection; then the cells were lysed with radioimmune precipitation assay buffer (150 mm NaCl, 1% Triton X-100, 1% sodium deoxycholate, 0.1% SDS, and 50 mm Tris, pH 7.5) supplemented with protease and phosphatase inhibitors on ice for 15 min. Lysates were then centrifuged at 12,000 × *g* for 10 min at 4 °C. Protein samples were mixed with 5× Laemmli sample buffer, and proteins were separated by SDS/PAGE, transferred to PVDF membranes, and incubated with primary antibodies overnight at 4 °C after blocking with 5% nonfat milk. Primary antibodies were detected with HRP-conjugated secondary antibody (Beyotime Biotechnology). For Co-IP experiments, 6 × 10^6^ cells were seeded in a 100-mm dish and harvested at 48 h posttransfection. The cells were then lysed in IP lysis buffer containing 50 mm Tris, pH 7.5, 150 mm NaCl, 1 mm EDTA, 10% glycerol, 1% Triton X-100, and protease and phosphatase inhibitors. Cleared cell lysates were incubated with the appropriate antibody (1–2 μg) overnight at 4 °C, followed by a 6-h incubation with protein A/G agarose beads at 4 °C. In all cases, the immune complexes bound to protein A/G beads were washed four times with IP lysis buffer and detached from the agarose using SDS loading buffer for immunoblot analysis.

The *in vivo* ubiquitylation assay was performed with hot lysis-extracted protein lysates based on the protocol previously described (47). Briefly, HEK293T cells were cotransfected with HA-Ub, FLAG-HIF-1α, and Myc-Tcf7l2, along with empty vector or VBP1-GFP. The cells were treated with 20 μm MG132 for 8 h before harvesting, then lysed in “hot SDS” lysis buffer (2% SDS, 150 mm NaCl, and 10 mm Tris-HCl, pH 8.0) with 2 mm *N*-ethylmaleimide and protease inhibitors, and lysates were boiled for 10 min and then diluted with nine volumes of dilution buffer (10 mm Tris-HCl, pH 8.0, 150 mm NaCl, 2 mm EDTA, and 1% Triton). The samples were incubated at 4 °C for 30–60 min with rotation and precleared by centrifugation at 12,000 × *g* for 5 min before performing immunoprecipitation.

### GST pulldown assays

*Escherichia coli* BL21 competent cells were transformed with GST or GST-VBP1 and then induced with 0.1 mm isopropyl β-d-thiogalactopyranoside at 37 °C for 5 h. The cells were collected by centrifugation, washed once with ice-cold PBS, and then lysed by sonication in lysis buffer (50 mm Tris, pH 7.5, 150 mm NaCl, 1 mm EDTA, 10% glycerol, and 1% Triton X-100) on ice. The GST or GST–VBP1 extract was then mixed with GSH Sepharose 4B Beads (GE Healthcare) overnight on a rotator at 4 °C. These mixtures were centrifuged, and the precipitates were diluted with an equal amount of indicated cell lysates of HEK293T and rotated for 2 h at 4 °C. After extensive washing with the lysis buffer, the precipitated proteins were eluted using SDS/PAGE. The bound proteins were detected by indicated antibodies or Coomassie Blue staining.

### BiFC and immunofluorescence

BiFC assays were performed as previously described (34). Venus fluorescence protein was selected as the reporter for complementation. Human VBP1 was fused with the carboxyl (VC, amino acids 155–239) terminal of Venus and then subcloned into the pBI bidirectional vectors. *Xenopus* Tcf7l1, Tcf7l1ΔN, and Tcf7l1ΔNLS were fused, respectively, with the amino (VN, amino acids 1–173) terminal of Venus. For immunofluorescence staining, 1 × 10^5^ cells were grown on coverslips inside a 6-well plate. After transfection, the cells were fixed with 4% paraformaldehyde, then permeabilized for 5 min at room temperature with PBS containing 0.25% Triton X-100 and blocked with 3% BSA in PBS. The cells were then incubated with anti-Myc antibody overnight at 4 °C. Goat anti-mouse Cy3 was used as a secondary antibody. After 4′,6-diamidino-2-phenylindole incubation, the cells were mounted in mounting medium. Images were acquired using a Leica SP8 confocal microscope.

### Generation of stable cell lines

For shRNA-transduced experiments, vectors named GV248 were purchased from Genechem (Shanghai, China). VBP1 shRNA-1 sequence 5′-GGAAGACCTTGACTTTCTTCGAG-3′ and VBP1 shRNA-2 sequence 5′-CAAGGATGACTCTACCAAGAACA-3′ were used. Lentivirus was produced in HEK293T cells. Briefly, 2 × 10^6^ HEK293T cells were seeded in a 60-mm dish with 4 ml of DMEM containing 10% FBS without antibiotic. Next day, pCMV-dR8.2 dvpr, pCMV-VSV-G, the lentivirus vector GV248 containing the GFP reporter gene and the puromycin resistance gene, and the shRNA sequence were cotransfected into the packaging cell line by Lipofectamine 2000. After 18 h, the transfection medium was replaced with fresh DMEM + 10% FBS, and medium containing the lentivirus was harvested after 72 h. Target cells were infected with filtered lentivirus; the infection was carried out in the presence of polybrene (8 μg/ml). At 72 h postinfection, 1.0 μg/ml of puromycin was added to the infected culture plates. The medium was changed every 2–3 days. Cells without integration of transfected plasmid died, whereas cells that had undergone plasmid integration survived at day 7 posttransfection. The surviving cells were allowed to expand in the culture plate, and when they reached a high confluence, these cells were cryo-preserved as a polyclonal line.

### CHX chase assay

Cells (6 × 10^5^) were seeded in 6-well tissue culture plates and then incubated overnight. After 12–18 h of incubation, the cells were cultured with CHX at a concentration of 100 μg/ml and incubated for various hours. The cells were harvested at the indicated time points. The lysates were prepared and subjected to immunoblotting analysis. The protein stability was determined by the percentage of TCF7L2 normalized to that of GAPDH at an indicated point compared with that at the initial point.

### Plate cloning formation assay

The colony formation assay was performed using lentivirus-infected HCT116 cells expressing either scramble shRNA or VBP1 shRNA. For each group, cells were plated at a density of 800 cells/well in a 6-well plate (Corning) and cultured in DMEM-high glucose supplemented with 10% FBS for 14 days. Culture medium was changed every 2 days. After most of the cell clones had expanded to >50 cells, the cells were washed with PBS and then fixed with 4% paraformaldehyde for 15 min, and staining was performed with 0.05% crystal violet for 20 min at room temperature. The excess crystal violet was washed with dH_2_O and then the dishes were allowed to dry. Clones were counted manually under a dissecting microscope. The statistical data of colony formation were expressed as mean ± S.D. from four independent experiments.

### Statistical analysis

All graphs were generated using GraphPad Prism5 Software. Values are presented as the means ± S.D. Statistical analyses were performed using a two-tailed, unpaired Student's *t* test or one-way analysis of variance followed by Tukey's post-test. A value of *p* < 0.05 was considered statistically significant. Unless otherwise indicated, all experiments contained three replicates per condition.

## Data availability

All the data arecontained within this article and in the supporting information. All the data are to be shared upon request (Jianfeng Zhou, Ocean University of China, jfzhou@ouc.edu.cn).
