# Supplementary material for: VBP1 modulates Wnt/β-catenin signaling by mediating the stability of the transcription factors TCF/LEFs
Source: J Biol Chem. 2021 Jan 13;295(49):16826–39. doi: 10.1074/jbc.RA120.015282 (PMC7864075; doi:10.1074/jbc.RA120.015282)
Supplement: Supplementary file 1 [file mmc1.pdf]

Sup Figure legends

Fig. S1 Amino acid sequence alignment of VBP1/Vbp1 and the spatiotemporal expression pattern of zebrafish *vbp1*. (A) Amino acid sequence alignment of human, mouse, *Xenopus*, and zebrafish VBP1/Vbp1. Conserved amino acids are indicated in black shadow. The accession numbers are as follows: human VBP1 NP_003363.1, mouse VBP1 NP_035822.2, Xenopus Vbp1 NP_001080849, and zebrafish Vbp1 NP_001018460.1. (B) RT-PCR analysis of *vbp1* mRNA in zebrafish embryos at indicated stages. (C) Whole-mount *in situ* hybridization analysis of *vbp1* transcripts at indicated stages. Scale bar, 200 μm. (D) Effectiveness of the used MO. One-cell stage embryos were injected with the *vbp1*-5'-UTR reporter plasmid DNA (50 pg) and control MO (cMO, 6 ng) or *vbp1*-targeting MO (MO1 and MO2, 4 ng and 6 ng). Fluorescence micrographs of injected embryos at 12 hpf are shown. Scale bar, 500 μm.

Fig.S1

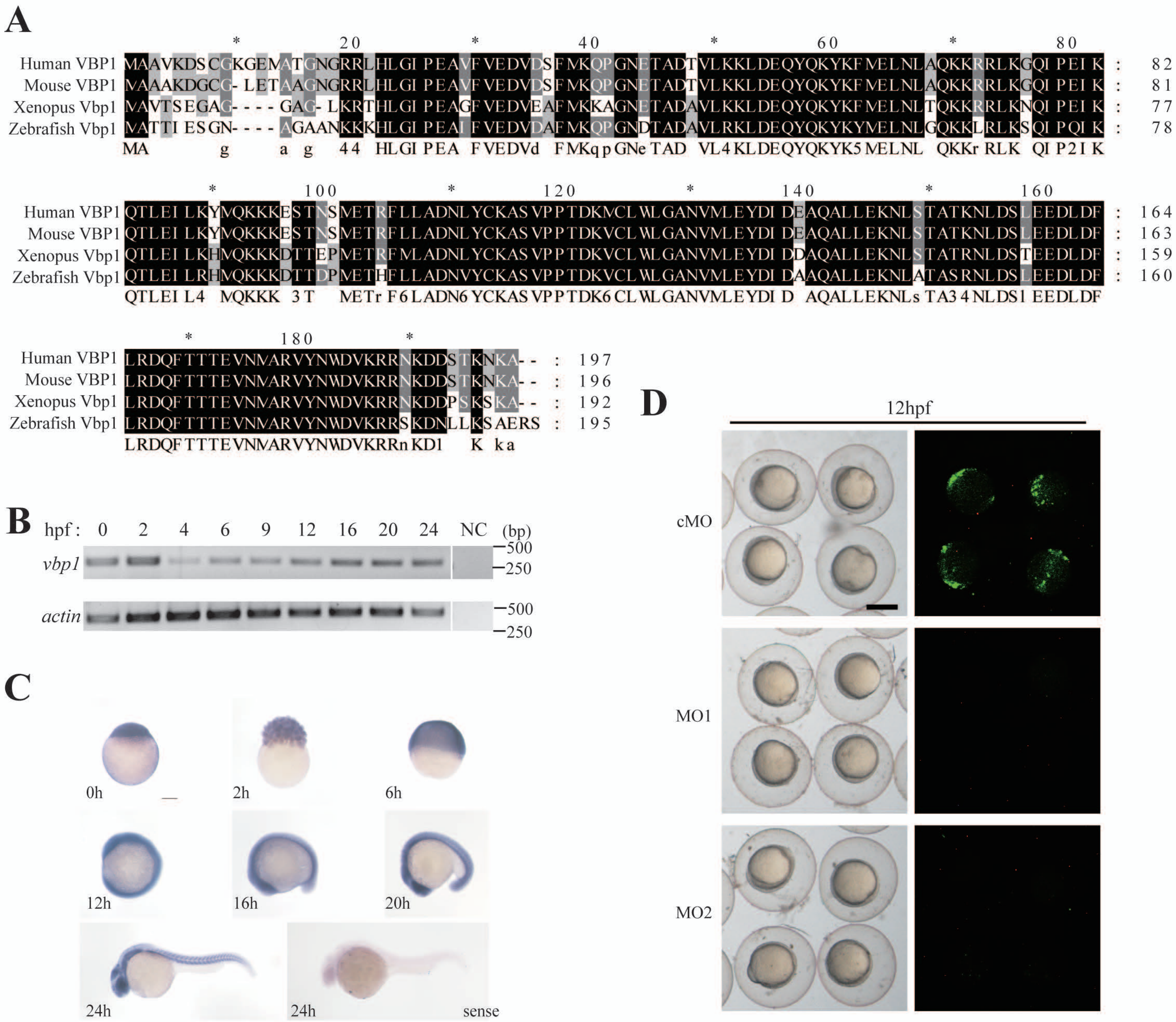
A
Human VBP1 MAAVKDSCGKGEMATGNGRRLHLGIPEAVFVEDVDSFMKQPGNETADTVLKKLDEQYQKYKFMELNLAQKKRRLKGQIPEIK : 82
Mouse VBP1 MAAAKDGCG-LETAAGNGRRLHLGIPEAVFVEDVDSFMKQPGNETADTVLKKLDEQYQKYKFMELNLAQKKRRLKGQIPEIK : 81
Xenopus Vbp1 MAVTSEGAG----GAG-LKRTHLGIPEAGFVEDVEAFMKKAGNETADAVLKKLDEQYQKYKFMELNLTQKKRRLKNQIPEIK : 77
Zebrafish Vbp1 MATTIESGN----AGAANKKKHLGIPEAIFVEDVDAFMKQPGNDTADAVLRKLDEQYQKYKYMELNLGQKKLRLKSQIPQIK : 78
MA g a g 44 HLGIPEA FVEDVd FMKqpGNeTAD VL4KLDEQYQKYK5MELNL QKKrRLK QIP2IK
Human VBP1 QTLEILKYMQKKKESTNSMETRFLLADNLYCKASVPPTDKMCLWLGANVMLEYDIDEAQALLEKNLSTATKNLDSLEEDLDF : 164
Mouse VBP1 QTLEILKYMQKKKESTNSMETRFLLADNLYCKASVPPTDKVCLWLGANVMLEYDIDEAQALLEKNLSTATKNLDSLEEDLDF : 163
Xenopus Vbp1 QTLEILKHMQKKKDTTEPMETRFMLADNLYCKASVPPTDKVCLWLGANVMLEYDIDDAQALLEKNLSTATRNLDSTEEDLDF : 159
Zebrafish Vbp1 QTLEILRHMQKKKDTTDPMETHFLLADNVYCKASVPPTDKVCLWLGANVMLEYDIDAAQALLEKNLATASRNLDSLEEDLDF : 160
QTLEIL4 MQKKK 3T METr F6LADN6YCKASVPPTDK6CLWLGANVMLEYDI D AQALLEKNLs TA34NLDS1EEDLDF
Human VBP1 LRDQFTTTEVNMARVYNWDVKRRNKDDSTKNKA-- : 197
Mouse VBP1 LRDQFTTTEVNMARVYNWDVKRRNKDDSTKNKA-- : 196
Xenopus Vbp1 LRDQFTTTEVNMARVYNWDVKRRNKDDPSKSKA-- : 192
Zebrafish Vbp1 LRDQFTTTEVNMARVYNWDVKRRSKDNLLKSAERS : 195
LRDQFTTTEVNMARVYNWDVKRRnKD1 K ka
B
hpf: 0 2 4 6 9 12 16 20 24 NC (bp)
vbp1
actin
500
250
C
0h
2h
6h
12h
16h
20h
24h
24h sense
D
12hpf
cMO
MO1
MO2
